# A New Structural Health Monitoring Strategy Based on PZT Sensors and Convolutional Neural Network

**DOI:** 10.3390/s18092955

**Published:** 2018-09-05

**Authors:** Mario A. de Oliveira, Andre V. Monteiro, Jozue Vieira Filho

**Affiliations:** 1 Department of Electrical and Electronic, Mato Grosso Federal Institute of Technology, Cuiabá 78005-200, Brazil; andrevmonteiro.eng@gmail.com; 2 São Paulo State University (UNESP), Campus of São João da Boa Vista, São Paulo 13876-750, Brazil; jozue.vieira@unesp.br

**Keywords:** SHM, electromechanical impedance, piezoelectricity, intelligent fault diagnosis, machine learning, CNN, deep learning

## Abstract

Preliminaries convolutional neural network (CNN) applications have recently emerged in structural health monitoring (SHM) systems focusing mostly on vibration analysis. However, the SHM literature shows clearly that there is a lack of application regarding the combination of PZT-(lead zirconate titanate) based method and CNN. Likewise, applications using CNN along with the electromechanical impedance (EMI) technique applied to SHM systems are rare. To encourage this combination, an innovative SHM solution through the combination of the EMI-PZT and CNN is presented here. To accomplish this, the EMI signature is split into several parts followed by computing the Euclidean distances among them to form a RGB (red, green and blue) frame. As a result, we introduce a dataset formed from the EMI-PZT signals of 720 frames, encompassing a total of four types of structural conditions for each PZT. In a case study, the CNN-based method was experimentally evaluated using three PZTs glued onto an aluminum plate. The results reveal an effective pattern classification; yielding a 100% hit rate which outperforms other SHM approaches. Furthermore, the method needs only a small dataset for training the CNN, providing several advantages for industrial applications.

## 1. Introduction

One of the most important and promising applications for structural health monitoring (SHM) systems is the aeronautics industry. Global aviation is growing rapidly promising even increased prospects for growth in the future. As discussed in [1] the world commercial aircraft fleet increased on average by 1.8% during 2010 reaching more than 25,000 new aircraft in operation. In addition, there are many old aircrafts that are still operating in the global air space [2,3]. It is important to mention that aged aircraft structures are predominantly made of aluminum and consequently monitoring the integrity of those metallic structures constitutes an increased demand for the aeronautics industry. Accordingly, new SHM methods may considerably reduce the maintenance cost and enable much more structural safety. Driven by new services in the area of structural analysis, SHM is developing in order to adapt academic work to practical SHM systems. Hence, NDE (Non-Destructive Evaluation) methods have extensively been proposed in recent years, as these methods allow the application of various types of structures and consequently the identification of various types of damage.

In SHM, physical or mathematical models are very useful for dynamic structural analysis, however, most of the real applications present some type of nonlinearity originated from geometric features, type of material, or boundary conditions, which leads to complex and expensive models. Owing to that, many authors have avoided working on structural modeling making use of techniques which allow the damage identification directly from the structural responses obtained from sensors signals. On this subject, the Electromechanical Impedance (EMI) technique plays an important role within the NDE methods. The EMI technique uses low-cost, small, and lightweight piezoelectric (PZT-lead zirconate titanate) transducer glued onto the structure [4]. This technique is well-known in the literature [5,6,7,8,9,10,11,12]. In the past, different SHM methods, based on neural networks (NN), had been widely investigated in order to evaluate structural conditions. Many SHM applications were proposed focusing on the multilayer perceptron and backpropagation algorithms [13,14,15]. Subsequently, new classes of NN such as probabilistic neural network (PNN) and fuzzy ARTMAP network (FAN) took place. These methods have been successfully shown on various structures. For example, methods based on PPN applied to damage identification in SHM were addressed in [16,17,18]. In the same way, FAN methods were investigated in [19,20,21,22,23,24,25,26,27].

More recently, the convolutional neural network (CNN) has exploded in popularity and real-world applications. The CNN simply provides a new class of NN which uses the concept of deep learning. CNN is one of the most recent major breakthroughs in the area of computer vision, speech recognition, biomedical systems, and natural language processing [28,29]. Unlike an ordinary NN, the layers of CNN can arrange neurons in three dimensions: width, height, and depth. Accordingly, some CNN applications have successfully emerged in the SHM field focusing mostly on vibration analysis as summarized next. Many CNN applications in SHM systems have focused on vibration analysis for monitoring faults on rotating machinery. For example, a fast and accurate motor condition monitoring and early fault detection system using 1D-CNN was proposed in [30]. Similar approaches were also addressed in [31,32]. Likewise, in reference [33] was proposed a method to address fault diagnosis based on CNN directly on raw vibration signals. The authors mentioned that the model works well in noisy environments and performs well when the working load changes. Similarly, a 1D-CNN vibration-based method was applied to damage detection and localization in real-time from the raw acceleration signals [34]. The method was applied to large-scale test structures. In [35], the authors proposed to incorporate sensor fusion by taking advantages of the CNN structure to achieve higher and more robust diagnosis accuracy. They analyzed both temporal and spatial information of the raw data from multiple sensors for the training process of the CNN. They pointed out that their method, compared with traditional approaches which use manual feature extraction, results in superior diagnosis performance. In [36], the authors proposed a CNN-based approach (LiftingNet) to learn features adaptively from raw mechanical data without prior knowledge. The authors highlighted that the advantages in applications are the ability to classify mechanical data sampled under different rotating speeds and achieving high classification accuracy with considerable noise present. Although all those above approaches obtained good results, none of them focused on using PZT-EMI-based method to identify structural damage. In [37], the authors proposed a wireless-sensor-networks-based method which takes advantage of an individual training 1D CNN for each wireless sensor in the network in a format where each CNN is assigned to process the locally-available data only, eliminating the need for data transmission and synchronization. That method operates directly on the raw ambient vibration condition signals without any filtering or preprocessing. In the same way, in reference [38], the authors proposed an enhanced CNN-based approach that requires only two measurement sets regardless of the size of the structure in order to overcome the limitation of training CNNs which predominantly requires a significant amount of measurements especially if applied to large-scale systems. They pointed out that their method was able to successfully estimate the actual amount of damage for the nine damage scenarios of the benchmark study.

Recently a single CNN application emerged in the SHM field focusing on video processing [39]. Therein, the authors proposed to analyze individual video frames for inspection of crack in a nuclear power plant via CNN and Naive Bayes Data Fusion. They pointed out that their framework achieves 98.3 of hit rate. Despite the good results, they mentioned that one disadvantage is that CNN needs substantial training data (e.g., more than 100,000 samples) to make the training converge and prevent overfitting. Another disadvantage is that the computations of CNN rely heavily on GPU (graphics processing unit). Conclusively, the SHM literature shows clearly that there are no details for the combination of PZT-EMI-based method and CNN when applied to monitor structures, underscoring the novelty of the approach presented here

Unlike existing studies, the major contribution of this work consists of a novel strategy for damage detection via the combination of the EMI-PZT-based technique and the CNN algorithm. The developed methodology was experimentally tested based on the EMI technique. The validation of the methodology was carried out in an aluminum plate which contains three attached PZT patches. The damage scenarios were simulated by gluing a small metallic nut at three different positions. The results, therefore, showed that it can identify various structural conditions with accuracy, reliability, and efficiency. In summary, the main contributions of this paper are:

- We developed a novel method suitable for mechanical data analysis. A method that takes advantage of the combination of the EMI-PZT-based method along with CNN.
- A way of converting PZT response based on the EMI technique to a RGB frame constitutes a novel approach;
- Frames were computed through a wide range of frequency instead of choosing only the best range in which the EMI presents higher sensitivity. This issue provides an important advantage because that task is very difficult;
- An unpublished frame dataset encompassing a total of four types of structural conditions for each PZT is introduced;
- An enhanced method which requires only a small dataset for training the CNN without using GPU. Furthermore, only three epochs are needed to yield 100% of hit rate.

The remainder of the paper is organized as follows. Firstly, the main theoretical fundamentals are addressed. Secondly, the developed method, highlighting the combination of the EMI-PZT along and the CNN algorithm, is presented. Next, the results followed by a comparison with other SHM approaches are presented. Finally, the paper concludes by highlighting remarks on the developed approach.

## 2. Theoretical Fundamentals

### 2.1. Structural Health Monitoring Systems

Structural health monitoring (SHM) systems have become a crucial element in maintenance and inspection activities in the industry, with special emphasis on aeronautical engineering, aerospace, civil, maritime and other related fields. Owing to the high level of safety required, the aeronautical industry has demanded high investments in order to guarantee an adequate operating condition in aircrafts. According to [40], SHM systems could significantly reduce maintenance costs, as the damage could be detected in early stage, accounting for 27% of the cost of its life cycle. In SHM, the damage is characterized by changes in the dynamic response of the structure due to variations in stiffness, mass, energy dissipation, mechanical impedance and/or geometric properties of the structure [41]. Hence, the concentration of various damages in a structure can lead to failures compromising the operation of the entire system. In general, the term “integrity” is the condition of the structure that allows its proper operation with satisfactory performance. In this context, structural integrity is the borderline condition between safety and failure of structural components [42].

SHM systems are characterized by their ability to detect, locate, quantify, and estimate the life of the structure according to the occurred damage [43]. However, according to [7] when incorporating smart materials (PZT, magnetostrictive strain, shape memory alloys, etc.) into the detection system, three more levels should be considered: self-diagnosis of structural damage, structural self-repair, and a simultaneous system of control and monitoring. In SHM, NDE (nondestructive evaluation) methods have extensively been proposed in recent years, as these methods allow the application of various types of structures and consequently the identification of various types of damage. NDE methods have been applied based on different techniques such as: acoustic emission, Eddy current, radiography, thermography, shearography, Lamb waves, and electromechanical impedance [42]. Wherein, the electromechanical impedance (EMI) technique plays an important role due to this technique makes use of a low-cost piezoelectric transducer (PZT) attached to the monitored structure [4]. In this technique, several structural responses are collected to evaluate the structure considering its dynamic condition through a forced excitation via PZT patches. It is remarkable that the same PZT is also used as a sensor to collect structural responses for further processing.

Considering the use of the EMI technique, piezoelectric materials play important roles due to these materials can be used as passive and/or active elements. These materials cover a large range of frequency (from a few Hz up to GHz). Low-frequency applications are covered mainly by the polycrystalline materials (ceramics, polymers or composites). In turn, crystals and thin films are the most used in high-frequency applications [44]. PZT ceramics have the following advantages: good electromechanical coupling, good stability, high stiffness, linear response to low-cost electric field [45]. Among the various types of piezoelectric materials, PZTs have shown very efficiently, being able to convert about 80% of the mechanical energy into electric energy [45].

From a practical point of view of applying the EMI technique in SHM systems, the PZT transducers are glued into the monitored structure by high stiffness adhesive glue based on cyanoacrylate or an epoxy resin. From that, a coupling is established between the structure and the transducer PZT enabling to monitor variations of the mechanical impedance of the structure by measuring the electrical impedance of the PZT [4]. Hence, exciting the PZT using a sinusoidal source V_X_ (with amplitude V_P_ and angular frequency (*ω*)) will produce a current I with amplitude I_P_ and phase Ψ. The electrical impedance of the PZT (Z_E_(*ω*)) is given as follows [4]:

(1) $$Z_{E}\left(\omega \right)=\frac{\mathrm{Vx}}{I}=\frac{1}{j\omega a}{\left({{\bar{\varepsilon }}_{33}}^{T}-\frac{Z\left(\omega \right)}{Z\left(\omega \right)+Z_{a}\left(\omega \right)}d_{3x}^{2}{\hat{Y}}_{xx}^{E}\right)}^{-1}$$

where Z_a_(*ω*) and Z(*ω*) represent the mechanical impedances for the transducer and monitored structure, respectively. In Equation (1), ${{\bar{\varepsilon }}_{33}}^{T},\;{\hat{Y}}_{xx}^{E},\;d_{3x}^{2}$, and j represent dielectric constant, Young’s modulus, electric field constant, geometric constant and imaginary unit respectively. Note from Equation (1) that any variation in terms of the structural impedance will cause changes in the electrical impedance of the PZT patch and this, in turn, causes changes in the EMI signatures. Extra details of how PZT impedance is related to the structural condition via the EMI technique can be explored in the following references [4,7,46,47,48,49].

### 2.2. The Convolutional Neural Network

The convolutional neural network (CNN) is a deep linear network inspired by the functioning of the visual cortex of mammals. Its first version was proposed by [50] and was conceived inspired by the work of [51]. Posteriorly, authors proposed an enhanced CNN architecture by incorporating processes of supervised learning through the backpropagation method [52]. In reference [53] was proposed the LeNet network, which can be considered the first architecture to present all features of the current CNN. Following Google’s involvement in the competition promoted by ImageNet, the largest database of image classification, CNN has become the state-of-the-art for image classification [54]. This made CNN popularity increase and, consequently, the amount of published work grew up proportionally. The main trend in the modeling of CNN is towards the use of ever deeper networks [54].

The fundamental difference between an “ordinary” neural network and a CNN consists of the fact that CNN uses the convolution operation instead of the multiplication of the array of neurons in at least one of its layers [55]. In the image processing, where the image is a two-dimensional matrix, the convolution operation is very useful for edge detection, image smoothing, attribute extraction, among other features. As a consequence, the convolution operation reduces the size of the original image due to the difference in the filter size. However, this reduction can be overcome by using the well-known zero padding technique.

There are three important distinctive features on CNN compared to other Neural Network (NN): shared weights, spatial/temporal subsampling, and local receptor fields [53]. The shared weight enables the network to learn only a smaller set of filters that can be applied to all the regions of the image, instead of learning specific weights for each region of the image, increasing the power of generalization of the network [56]. The subsampling procedure in the CNN is usually conceived in the pooling layer (downsampling). This concept was first introduced by [52]. For that, the max pooling computation is done for an image region followed by creating an array of these maximums. Thus, it eliminates non-maximum values, reducing both the size of the data representation and the computation required for the next layers [55].

The third distinctive feature is the existence of local receptor fields. In the classical NN, each input value of each layer is completely connected to the input values of the previous layer (fully connected). Hence, the NN needs to perform several multiplications to find the connected neuron activation, requiring a great computational power mainly for images that have many connected neurons. On contrary, as in natural images, the adjacent pixels tend to be more strongly correlated than the distant pixels, the CNN is architected for that each filter learns on only one subregion of the data received from the previous layer [56]. This allows increasingly complex patterns to be modeled from combinations of simple local operations [55]. In addition to these important properties, other computational resources are used to avoid overfitting and training time of CNN. For example, the dropout consists of randomly removing half of the neurons from the hidden layers at each iteration of the training procedure. This technique also gives the network the ability to learn more robust parameters, since a neuron cannot depend on the specific presence of other neurons.

**Figure 1:**
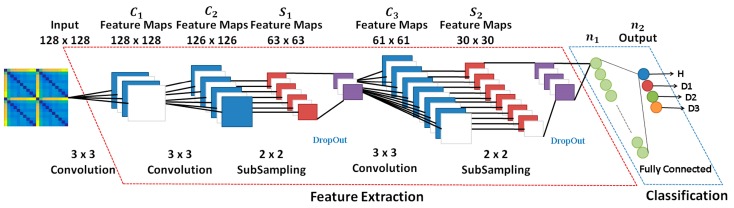
A general architecture for the CNN highlighting the layers.

In summary, CNN networks are composed of convolution layers, which involve the convolution process and the pooling process, in addition to using the concept of local receiver fields to optimize the image processing; layer normalization, which involves the dropout process and other processes used to improve network performance; and the fully connected layer responsible for sorting. Figure 1 shows a general architecture for the CNN. The first part of the network consists of the convolution (C1, C2, etc.) and subsampling (S1, S2, etc.) layers. Basically, these layers are responsible for extracting the network features. The second part of the network consists of the normalization and fully connected layers. This block is used as images classifier after the image has passed through the feature extraction block. The data entries of each hidden layer form a set of feature maps obtained by processing the data in the previous layer. The feature maps do not require the preprocessing of the image, which is a process that usually requires higher computational power, playing a fundamental role in the advantage of the use of this type of network in image processing. Extra details about CNN and deep learning are shown in previous studies learning [50,51,52,53,54,55,56,57,58].

## 3. Developed Method

**Figure 2:**
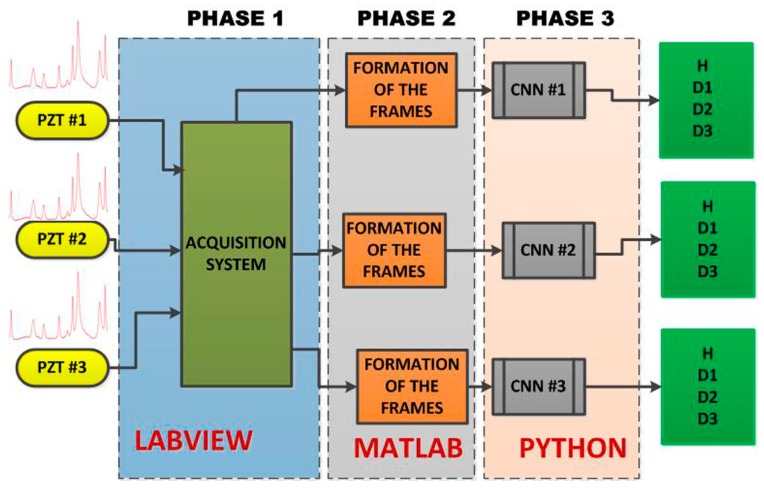
Developed framework for structural damage detection, based on the CNN algorithm, including all three phases.

The Figure 2 shows the developed framework for the methodology based on the EMI-CNN applied to identify structural damage. The methodology consists of three phases as described in the following subsections. In phase 1, impedance signals are obtained based on the EMI principle. For this, three PZTs (called PZT#1, PZT#2 and PZT#3) considering four different structural conditions (Healthy (H), Damage 1 (D1), Damage 2 (D2) and Damage 3 (D3) were considered. Further details about the experimental set up are presented in the next subsection. In phase 2, Euclidean distances (ED) were computed from the structural response signals in order to form frames. Those frames were used to form a dataset for both the training and test phases. In phase 3, the dataset was used as inputs for the CNN. Each CNN is responsible for recognizing four different structural conditions: H, D1, D2, and D3.

### 3.1. Phase 1: Acquisition of the EMI Signals

**Figure 3:**
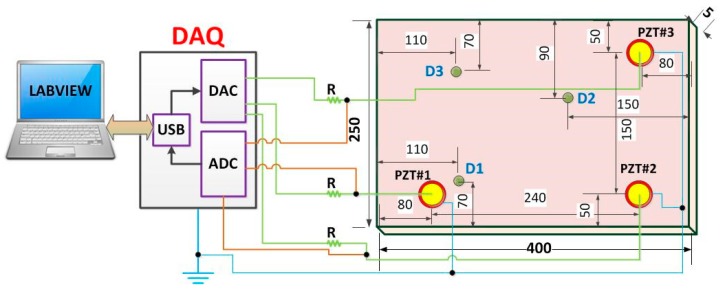
Representation of the general diagram for the acquisition system (dimensions in millimeters) [27].

In order to obtain the structural response signals, we developed a method based on the EMI technique. EMI requires that the structure is excited through a PZT at low amplitude considering over a wide frequency range to produce a forced excitation of the structure [4]. Each PZT acts as actuator and sensor at the same time. In our example, an aluminum plate of size 400 mm × 250 mm × 5 mm was suspended in both tips using fishing lines in order to simulate free-free boundary conditions. Three piezoelectric diaphragms (called PZT#1, PZT#2 and PZT#3) with diameters of 12 mm were used, that had active elements of type P-7 PZT ceramics (Murata Electronics). These diaphragms were bonded (using 3M Scotch-Weld Epoxy Adhesives DP460 Off-White) to the plate at three different positions (Figure 3).

Subsequently, a chirp signal sweeping from 20 kHz to 110 kHz with amplitude of 3 V was used to excite the set PZT/structure. Although many authors consider that the real part of EMI in a frequency range from 20 kHz up to 40 kHz constitutes the best set in terms of damage sensibility (for example [6,7]), the frequency band of the EMI signature for higher sensitivity and repeatability depends on several features, such as geometry, mass, boundary conditions and other structural features [5]. Also, studies show that the structure suffers less interference of global conditions in higher frequencies vibration modes [10], which justify the chosen frequency range. Another important remark regarding the excitation signal is that its variation in terms of amplitude does not affect the EMI-signatures [59].

**Figure 4:**
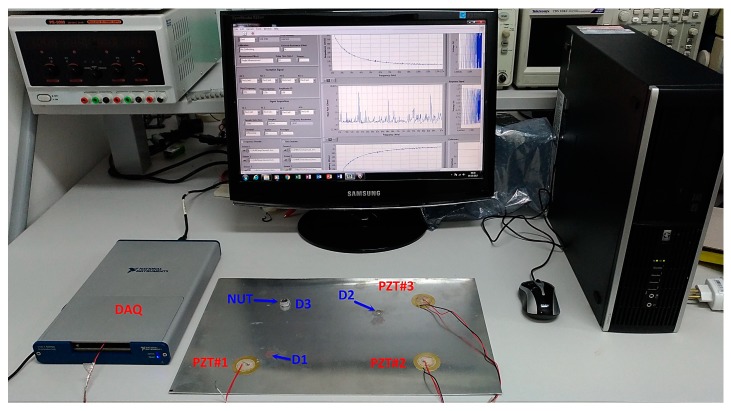
Experimental set up including: aluminum plate containing three PZT patches, DAQ (Data Acquisition) and computer running the acquisition software [27].

The acquisition system (DAQ) was developed in LabVIEW software and used here to excite and obtain the structure responses [8]. This system is pictured in Figure 4. The resistor R was set to 1 kΩ, in order to limit the electric current through the PZT patch. Using that system, a set of measurements for the pristine structural condition was performed. These measures were stored to form the Baseline (B) set. Each PZT response signal was separately sampled at a rate of 1 MS/s. At a different time, a new set of measurements, considering the same structural condition, was carried out to form a new data set for the Healthy (H) condition.

Next, three damage cases were separately simulated by gluing (using 3M Scotch-Weld Epoxy Adhesives DP460 Off-White) a metallic nut of about 10 g (diameter of 12 mm and height of 7 mm) at three different positions in the structure (Figure 2 and Figure 3), being only one damage per time (named D1, D2 and D3). Hence considering D1, the PZT#1 was separately excited and its own response is obtained individually, as proposed in references [4,5,6,7,8]. Afterward, the same procedure is applied to PZT#2 followed by PZT#3. From this approach, the response signals are obtained separately for each PZT patch, thereby allowing the proposed method to work on each response signal separately. Posteriorly, the nut was removed and bonded at the position D2. The response signals for PZT#1, PZT#2 and PZT#3 were separately obtained. Finally, the same procedure was carried out for D3. In a total, there were 1080 EMI signatures (60 for each structural condition). The time interval between two consecutive samples was 30 s. The environmental temperature of the room was kept constant to 22 °C throughout the experiment. The EMI signals were used to form RGB frames.

### 3.2. Phase 2: Formation of the Frames

**Figure 5:**
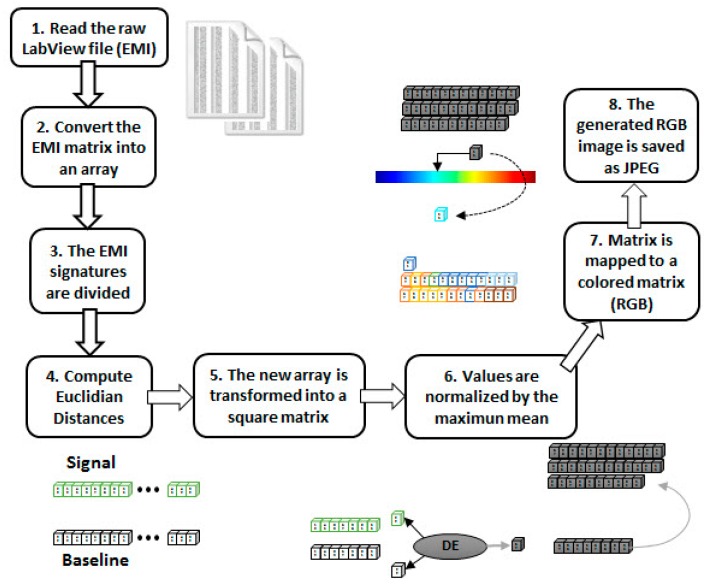
Block diagram for generating a RGB frame.

As stated earlier, the SHM literature shows clearly that there is not a combination of PZT-based methods and CNN due to the difficulty in obtaining images/videos from the PZT responses. As a consequence, there is a lack of using CNN along with the EMI technique applied to monitor structures. To overcome that, this paper introduces an innovative way of forming frames from PZT-EMI signatures, as explored in detail next. The RGB frame formation process is composed of eight steps, as follows (Figure 5):

- **Step 1:** The matrix containing the raw EMI data, sampled by the LabVIEW acquisition software, is read;
- **Step 2:** As the proposed method uses only the real part of the EMI, those samples are retrieved from the matrix into an array;
- **Step 3:** The EMI signatures (baseline and unknown conditions) are divided into equal parts (10 parts for each signal);
- **Step 4:** Those parts are used to compute Euclidian distances and generate a new array;
- **Step 5:** That new array is transformed into a square matrix;
- **Step 6:** Those obtained values (inside the array) are normalized by the maximum mean;
- **Step 7:** Using the *colormap function* (MATLAB), the normalized matrix is mapped to a colored matrix (RGB);
- **Step 8:** The generated image is then saved as a JPEG image. The image will be used as an input to the CNN preprocessing block (Figure 9).

**Figure 6:**
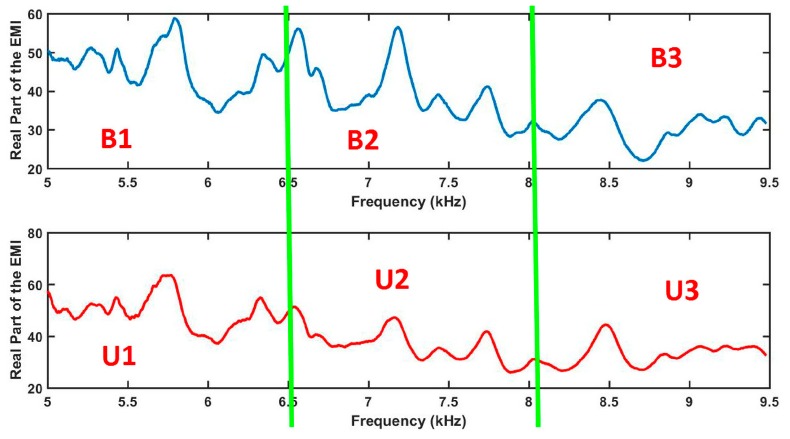
Division of the EMI signals for the baseline (top) and unknown (bottom) structural conditions before computing ED.

Next, the most important steps are further detailed from a practical point of view. Firstly, the real parts of the EMI are divided into several parts as illustrated in Figure 6 (Step 3). For example, Figure 6 shows two EMI signatures for the baseline (top) and unknown (bottom) conditions. Each signal was equally divided in three parts forming six parts in a total. Those parts were named as B1, B2 and B3 for the baseline signature and U1, U2 and U3 for the unknown condition.

**Figure 7:**
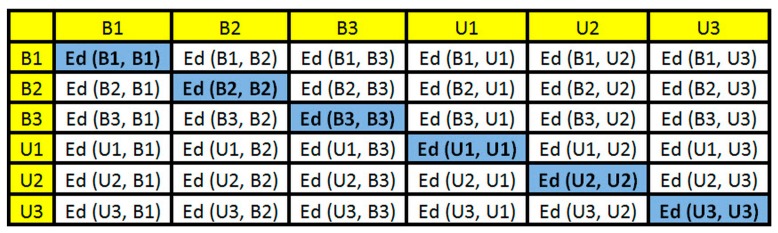
ED-matrix formed after computing ED from the EMI signatures.

Using MATLAB^®^, Euclidean Distances (ED) were computed from the EMI parts, as follows (Step 4):

(2) $$\mathrm{Ed}\left(B1,B1\right)=\sqrt{\overset{n}{\underset{j=1}{\sum }}{\left(B1_{j}-B1_{j}\right)}^{2}}$$

(3) $$\mathrm{Ed}\left(B1,U1\right)=\sqrt{\overset{n}{\underset{j=1}{\sum }}{\left(B1_{j}-U1_{j}\right)}^{2}}$$

where, B1 and U1 are the baseline and unknown structural conditions, respectively. This procedure was repeated among all parts in order to form an ED-matrix. Considering the example case, Figure 7 sums up all possible combinations of the ED into an ED-matrix (Step 5).

**Figure 8:**
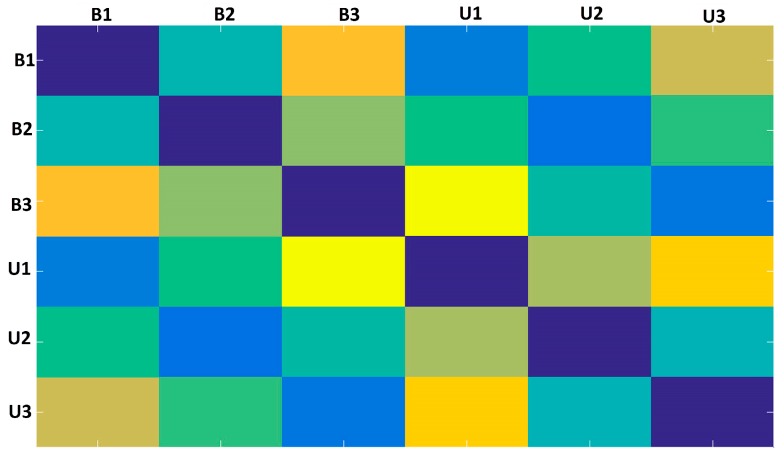
Obtained frame from two random PZT-EMI signatures.

It is important to highlight that the principal diagonal of the ED-matrix is zero because the method computes EDs for the same part of the signals there. This matrix is formed for each PZT-EMI signature and this will be used to form a frame. In this paper, the baseline signature is always used in the first part of the ED-matrices. Each element of ED-matrix was transformed into a RGB (red, green, and blue) scale in order to form a RGB frame with three dimensions (width, height, and depth). This procedure was easily run in the developed MATLAB software (Step 7). Figure 8 shows its correspondent RGB frame for the previous example (Figure 7). Each obtained frame has a width, height, and depth of 895, 656 and 3, respectively.

As observed in Figure 8, the obtained frame presents regular symmetry over and under the principal diagonal. Once the structural condition varies, the frame colors will turn accordingly. As a consequence, each frame will be subtlety different for each structural condition and such differences will be perceived by the CNN algorithm. It is important to mention that during the frame assembly, we form a corresponding frame for each PZT-EMI signature along with its respective baseline signature. Furthermore, the developed methodology assembles frames through a wide frequency range instead of choosing only the best range in which the EMI technique presents higher sensitivity, as is the case in standard EMI approaches. This is an advantage because it eliminates the difficult task of searching for the most sensitive frequencies [60]. From the assembled frames a frame dataset with 720 frames formed from the EMI-PZT signals, encompassing a total of 4 types of structural conditions for each PZT is formed. Table 1 shows how the dataset is distributed for PZTs #1 and #2. The distribution for PZT#3 is similar. This dataset is used as input to feed the CNN algorithm.

### 3.3. Phase 3: CNN-Based Damage Detection Method

As aforementioned, the CNN forms a new class of neural networks (NN) which uses the concept of deep learning [50,51,52,53,54,55,56,57,58]. The CNN takes advantage of the fact that the input consists of images/videos and they constrain the architecture in a more sensible way. Unlike an ordinary NN, the layers of a CNN have neurons arranged in three dimensions: width, height, and depth. According to [57], the CNN architecture was designed to ensure some degree of shift, scale, and distortion invariance. Further, each unit in a layer is organized in planes which all units share the same set of weights. The set of outputs of the unit in a given plane is called a feature map. Hence, a full convolutional layer is composed of several feature maps with different weight vectors. As a consequence, several features can be extracted at each location in the image [57]. A sequential implementation of the feature maps consists in scanning the image with a single unit that has a local receptive field and stores the states of this unit at the corresponding position on the feature map. The kernel (filter) of the convolution process is used to connect weights used by the units into the feature maps [57]. It is fair to say that the recent success of the CNN architecture can be largely attributed to the strong emphasis on modeling multiple levels of abstractions.

**Figure 9:**
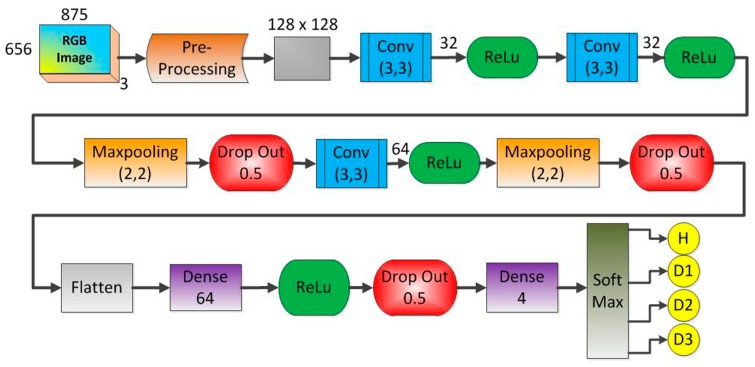
Architecture of the developed CNN to identify structural damage.

In order to evaluate structural conditions, this approach proposes a framework for the CNN as shown in Figure 9. The method uses one CNN architecture like that for each PZT sensor. The CNN is fed with the obtained frames computed from the impedance signatures under various structural conditions (Table 1). The preprocessing block is the first step to be considered. This block consists of two steps of image processing. The first step is to read and convert the RGB image to grayscale. Besides, the image is resized from 875 × 656 × 3 pixels to 128 × 128 × 1 pixels in order to reduce the processing time for the CNN. A second step towards finalizing the preprocessing block consists of converting the grayscale image into a feature vector by flattening the image to an array. This array contains all the pixels of the image and it is structured by adding the first row of pixels from the image to an empty array, then, the second row of pixels is added to the end of that array and so on, until the last row of the image. Posteriorly, the array type is changed to float to enable performing of standardization. This process is important because some machine learning algorithms may present low performance when there are large variations in the used data. Finally, the array is normalized with Gaussian distribution with zero-mean and unit-variance.

A brief explanation of the most significant characteristics of the CNN architecture shown in Figure 9 is stated next. Firstly, the grayscale image [128 × 128 × 1] was applied to the first Conv module. This module is composed of 32 filters (kernel size of 3 × 3), resulting in a volume such as [126 × 126 × 1]. It is important to mention that this Conv block applies the zero-padding algorithm aiming to avoid losses. Other Conv modules can be understood similarly except they do not take advantage of the zero-padding algorithm. Next, the Maxpooling size was set to 2 × 2 whilst the Drop Out was set to 0.5 in order to reduce the possibility of over-fitting and improve the generalization of the CNN. In order to optimize the weights of the training algorithm, this approach used the popular RMS (Root Mean Square) backpropagation algorithm wherein the weights were changed according to the gradient descent direction of an error. The Soft Max block outputted four structural conditions: H, D1, D2, and D3.

## 4. Experimental Results

**Figure 10:**
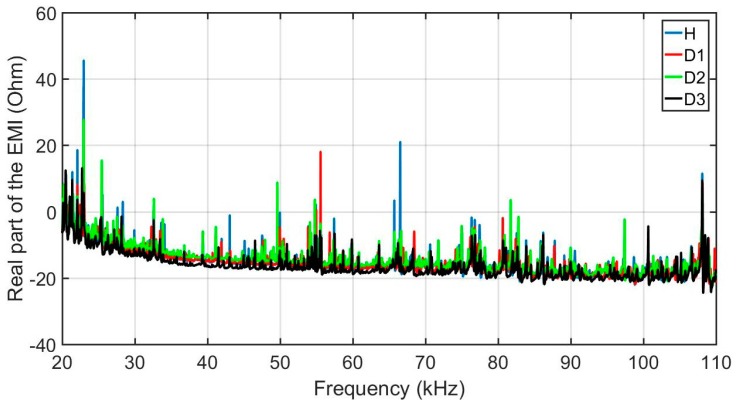
Real part of the EMI, for PZT#2, considering various structural conditions (H, D1, D2 and D3).

In order to evaluate the developed methodology, this section presents the results obtained considering the experimental set up described above. Firstly, structural response signals were obtained from the various structural conditions and positions of damage on the structure, through PZTs using the above-mentioned acquisition system. Sample signals are presented in Figure 10. For brevity, only the real part of the impedance for PZT#2 is shown. Signatures are shown for four different structural conditions: healthy (H), damage 1 (D1), damage 2 (D2) and damage 3 (D3). As observed, the damage insertion will cause changes in the electrical impedance of the PZT and this in turn causes changes in the EMI signatures. Those changes happen in both magnitude and frequency. For the majority of the cases, the structural change causes only subtle variations in the EMI signatures needing methods more precise that are able to identify such variations automatically.

**Figure 11:**
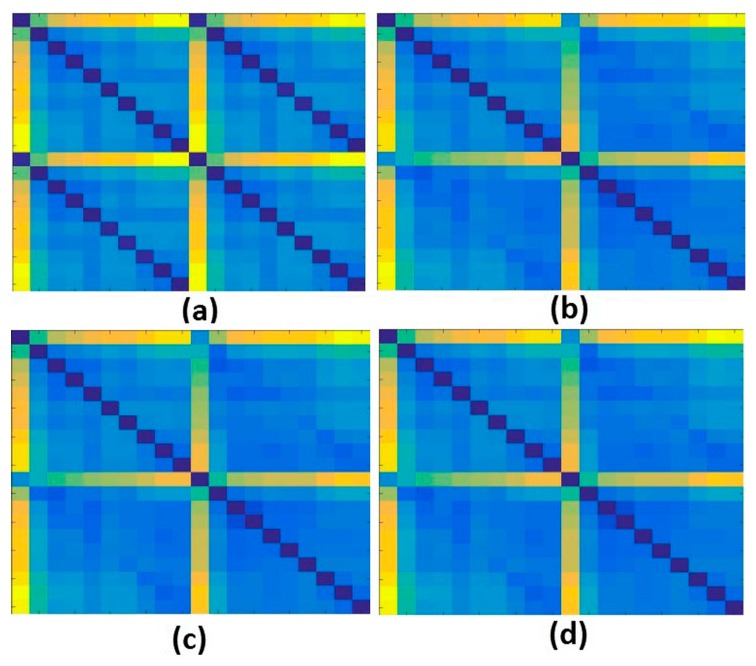
Set of frames formed from the EMI signatures for PZT#2: (**a**) baseline with Healthy (H); (**b**) baseline with damage 1 (D1); (**c**) baseline with damage 2 (D2); (**d**) baseline with damage 3 (D3).

Secondly, the obtained structural response signals were divided, in ten parts for each signal, as shown in Figure 6. Next, Euclidean distances (ED) were computed from the split signals, as in Equations (2) and (3) and as following those values are put onto the ED-matrix (Figure 7). From the formed ED-matrix, each ED value is transformed to the RGB frame (see procedures presented in Figure 5). Figure 11 illustrates a set of frames formed from the EMI signatures for PZT#2.

The frame showed in Figure 11a is formed by computing ED for signatures for the baseline with healthy (H) structural conditions. Similarly, Figure 11b–d present the formed frames considering the baseline with D1, baseline with D2 and baseline with D3, respectively. As observed in Figure 11, a substantial difference between the frames for healthy and damaged conditions is perceptible, mainly from the second diagonal. This diagonal is only presented for the healthy condition. There are also subtle differences, almost imperceptible by human eyes if we analyze the frames brightness. In contrast, taking into account only the frames for the damaged structural conditions, such differences are visually misperceived demanding a very precise algorithm to overcome that. In this sense, this approach applies the CNN algorithm to bring up those subtle differences in order to provide precise and reliable damage detection, as shown next. It is important to highlight that the obtained frames are used to form a dataset for training and testing procedures, which are used as input to the CNN algorithm (Table 1).

Thirdly, the CNN block is fed to the aforementioned dataset (Table 1). Both training and testing phases were carried out on a Laptop running Windows 8. The Laptop has an Intel Core i5-3320 M with 8 GB of RAM. It is important to point out that this approach did not take advantage of any dedicated GPU. Keras along with Theano backend libraries were used to run the training and test. Those libraries were specially developed in Python for deep learning applications. The batch size was set to 8 and, after running 3 epochs the training procedure successfully converged. Three CNN blocks were designed, corresponding one for each PZT sensor.

**Figure 12:**
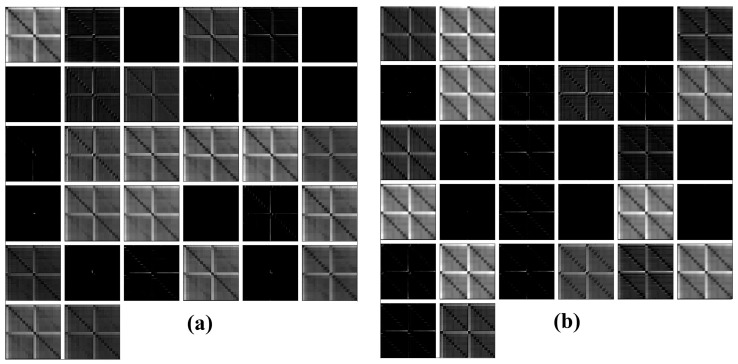
Feature maps for the 1st CNN layer after applying 32 kernels into PZT#2 frames for the structural conditions: (**a**) D1; (**b**) H.

Figure 12 shows the feature maps for the 1st CNN layer after applying 32 kernels onto a correspondent frame for PZT#2, considering D1 and H structural conditions. Analyzing Figure 12, we can realize that there are substantial differences among the feature maps for D1 and H. Such differences are primordial to guarantee the suitability of the developed methodology. In order to extract the most relevant features of the frame, each frame is passed by several blocks: Conv, Maxpooling, ReLu, Drop Out and so on (Figure 9).

**Figure 13:**
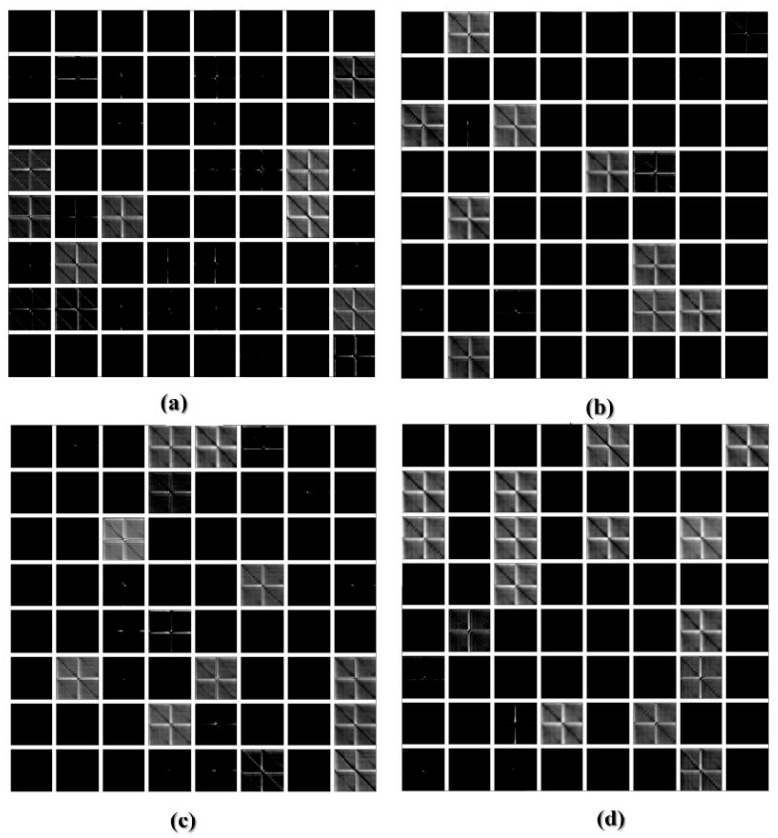
Feature maps for the 7th CNN layer after applying 64 kernels into PZT#2 frames for the structural conditions: (**a**) Healthy (H); (**b**) D1; (**c**) D2; (**d**) D3.

Figure 13 depicts the output (feature maps) for the third Conv block (7th CNN layer) in the framework (Figure 9), after applying 64 kernels onto PZT#2 frames considering H, D1, D2 and D3 structural conditions. Investigating the results presented in Figure 13 it is possible to see how PZT#2 perceives each structural condition. Further, it is clear that each frame presents outstanding distinctive features for each structural condition compared with the results presented in Figure 11, therefore, making this methodology very promising in SHM.

Considering that one CNN is designed for each PZT sensor and the training and testing phases have been carried out, the CNN successfully converged after running three epochs. Table 2 shows separately the results for each PZT. The results show that the method was effectively able to identify various structural conditions with 100% accuracy. It is important to highlight that this result was obtained using only a small dataset for training the CNN (Table 1) without using any type of GPU. This may provide an excellent and reliable solution for industrial applications where the availability of structural response signals to form the training set is generally scarce.

**Figure 14:**
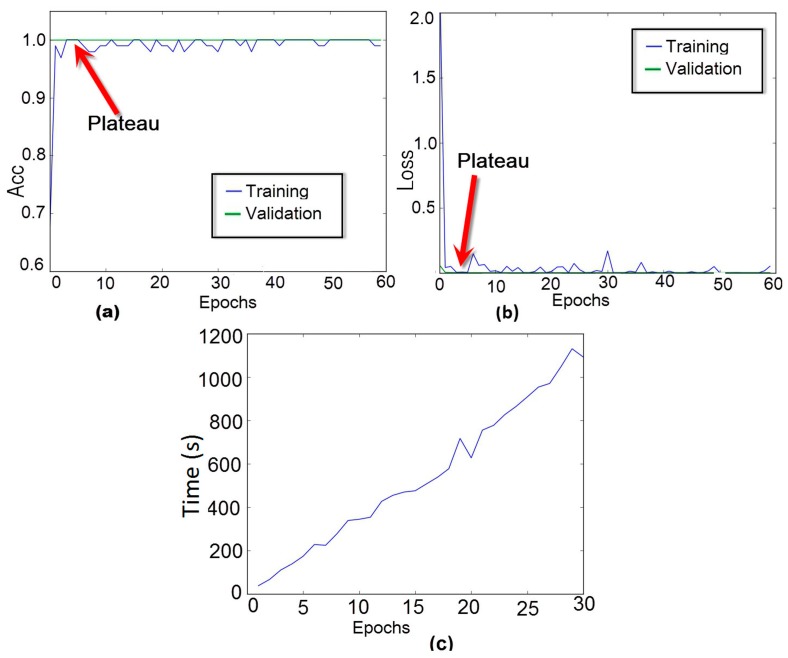
Performance analysis of the CNN for PZT#2: (**a**) training and validation accuracy curve of the model as a function of epoch; (**b**) training and validation loss curve of the model as a function of epoch; (**c**) Consumption time versus number of epoch for the training phase.

As stated earlier, the developed method successfully converged after running 3 epochs. This issue is further investigated in Figure 14. For that, the method was evaluated varying the number of epochs from 1 to 60 and, the accuracy and loss rates (for PZT#2), for both training and validation phases, were computed and presented in Figure 14a,b, respectively. From the results, we can see that during the validation phase the accuracy rate was always constant and equal to 100%. On the other hand, during the training procedure, this rate shows significant variations. However, there is a small plateau for the third epoch. A similar analysis can be done for the loss rate showed in Figure 14b. The method results in a loss rate of zero for three epochs. Based on these results, we henceforth set the number of epochs to three. It is fair to mention that the number of epoch has a straight relation to the training times as shown in Figure 14c. Analyzing Figure 14c we can realize that when using three epochs, the training time is about 120 s.

## 5. Comparison with Other State-of-the-Art Solutions

In order to evaluate the performance of the developed method, Table 3 shows the success rates for testing phase as a comparison of different methods, running in the same conditions. Methods based on probabilistic neural network (PNN) [24], simplified fuzzy ARTMAP network (SFAN) [22,24], Savitzky–Golay (SG), Savitzky–Golay with first derivative (SGFD) and Savitzky–Golay with second derivative (SGSD) were considered [27]. The SFAN, SG, SGFD and SGSD methods used setup parameters as follows: ρ = 0.78, α = 0.25 and β = 1 [24,27]. For the PNN, the spread constant (σ) was set to 0.1 [24,27]. Analyzing Table 3, we can realize that the method enhanced the success rates for all PZTs sensor. For example, PZT#1 yielded an improvement of 17% and 6% compared with the SFAN-SGSD and SFAN-SGFD methods, respectively. Therefore, the enhancement of this approach over existing approaches is undoubted.

Training and testing times are stated next. As aforementioned, the training time is directly related to the number of epochs. Hence, Table 4 shows a time consumption comparison for three different methods, considering the results for PZT#1. All methods were run, under the same conditions, on a laptop (stated earlier).

As observed in Table 4, the SFAN-based method showed the best performance in terms of both training and testing times. The PNN method obtained the second place with a subtle difference in relation to SFAN. The CNN-based method results in a longer time for both training and test. This is because the processing images consist of a time-consuming task as recurrently shown in the literature. Further analysis about time consumption will be stated in the next subsection.

### Advantages and Drawbacks

The feasibility of the above approach is validated based on EMI-measurement datasets. The method results in an accuracy rate of 100% for all tested scenarios. Therefore, the main advantages of the developed method can be summarized into four points.

Firstly, a new way of converting PZT response to RGB frames along with the CNN-based method represents a new approach to structural health monitoring. Based on the results, the method has direct implications in terms of diminishing the percentage of false alarms whilst the damage detection is being performed.

Secondly, the major achievement in applying the method is the ability to classify structural damage with higher accuracy compared with the state-of-the-art approaches [22,24,27]. This is possible because the CNN applies several banks of filters in order to extract the best features that represent different structural conditions, in each frame. It potentially has direct application in the composite materials industry especially when applied to identify small damage and its progression as discussed in [22,24,61].

Thirdly, it is important to quote that this method, proves to be more reliable to detect both internal and non-visual damage compared with a method based on only video/image processing [39]. Furthermore, the method presents another important advantage compared with [39] because it does not require a GPU and can be run in an ordinary laptop, a direct consequence of the small dataset used to train the CNN.

Fourthly, the developed method forms frames through a wide range of frequency instead of choosing only the best range in which the EMI presents higher sensitivity. This issue comprises an important advantage because that task is very difficult as pointed out in [62].

Despite the advantages, improvements of the developed method still need to be investigated. The major problem with the current approach is the time consumption issue. As presented in Table 4, the CNN-based method used substantially more time compared with the methods addressed in [22,24,27]. On the other hand, it is important to mention that if we essentially consider that the majority of the industrial applications run the training phase offline and, that the demanded test time is 7.93 s for processing 96 frames, thus it would take a meaningfully time of 83 ms for processing one single frame. This time seems to be impeding for real-time applications; however, in practice, the procedure of acquisition of the EMI signatures can be carried out in an even longer time frame (e.g., over minutes). Therefore, the method can be perfectly considered for many SHM applications running on real-time using an ordinary laptop.

It is important to remark that SHM techniques based on PZT transducers are highly influenced by environmental conditions, noise [33,36,63] and especially temperature, during structural damage detection procedure. There are several approaches to compensate temperature effects on PZT-based SHM systems and most of them make corrections in the EMI signatures [49,64,65]. So, although the proposed method does not consider temperature variations, under such conditions it will only requires a preprocessing of EMI signatures before using them, which does not change the proposed method.

To date, we can only guarantee the capabilities of the method for damage detection and size/type estimation in terms of a rather large size as defined here. Smaller damage sizes will form future investigations. However, the simulated damage represents an only negligible increment of structural mass when compared to the total mass of the structure, which is compatible with real damage. Furthermore, future research will be undertaken to evaluate the accuracy of the developed method for randomly initiated defects and to establish the outcomes from having two or more areas of damage at the same time. Another interesting point to be addressed is the evaluation of different type, position, and size of damage [66,67,68].

## 6. Conclusions and Future Work

This paper has introduced an exploration of the suitability of a CNN-based method applied to monitor structural damage in aluminum structures. Accordingly, we developed a method which takes advantage of the combination of the EMI-PZT-based method and CNN. This methodology presents a new approach for SHM. Additionally to the fact of that CNN-based method was developed here for the first time, this approach consists of a reliable and innovative way of converting PZT response based on the EMI technique to the RGB frame.

Based on the results, the CNN-based method shows significant enhancement in terms of the overall success rate whilst the structural damage detection is carried out. As a result, a hit rate of 100% was obtained running only three epochs, which outperforms current approaches. Furthermore, the method runs only a small dataset for training the CNN without using any type of dedicated GPU. To conclude, the method identified the damage scenarios with higher accuracy, therefore, rendering this approach in a promising and useful contribution in the SHM area.

Future work will focus on the evaluation of the sensitivity of the developed methodology to identify the progression of the structural damage in structures made of composite materials. Those materials present a higher damping coefficient compared with aluminum, demanding a more accurate method such as shown throughout the paper. Future goals, motivated by the outcomes presented in this paper, will focus on features other than the success rate for measuring the classifier efficiency such as the Kappa coefficient. Further research will be undertaken in evaluation the CNN configuration such as: image size, training dataset size, and the number of kernels to optimize the required time consumption.

## Figures and Tables

**Table 1:** Distribution of number of frames, formed from the PZT-EMI signals for PZTs #1 and #2, into the dataset.

Structural Conditions	PZT #1	PZT #2		
	Training	Test	Training	Test
Healthy (H)	36	24	36	24
Damage 1(D1)	36	24	36	24
Damage 2(D2)	36	24	36	24
Damage 3(D3)	36	24	36	24
Total	144	96	144	96

**Table 2:** Results for the CNN method: training and testing phases.

Sensors	Training Accuracy	Testing Accuracy
PZT #1	98%	100%
PZT #2	100%	100%
PZT #3	100%	100%

**Table 3:** Comparison of the CNN-based method with other NN approaches: Success rates obtained for the testing phase.

Methods	PZT#1	PZT#2	PZT #3
CNN	100.00%	100.00%	100.00%
SFAN-SGSD [27]	83.33%	100.00%	98.95%
SFAN-SGFD [27]	94.79%	85.41%	88.54%
SFAN-SG [27]	83.33%	100.00%	98.95%
SFAN-ED [22,24]	61.41%	98.95%	77.08%
PNN-SGSD [27]	75.00%	100.00%	98.95%
PNN-SGFD [27]	50.00%	75.00%	85.41%
PNN-SG [27]	75.00%	100.00%	98.95%

**Table 4:** Comparison among consumption times for: CNN, PNN, and SFAN.

Methods	Training Time (s)	Testing Time (s)
CNN	121.10	7.9300
SFAN [22,24,27]	0.1265	0.0079
PNN [24]	1.6724	0.6742
